# MiR-130a-3p Alleviates Liver Fibrosis by Suppressing HSCs Activation and Skewing Macrophage to Ly6C^lo^ Phenotype

**DOI:** 10.3389/fimmu.2021.696069

**Published:** 2021-08-05

**Authors:** Lei Liu, Peng Wang, Yun-Sheng Wang, Ya-Nan Zhang, Chen Li, Zi-Yin Yang, Zi-Hao Liu, Ting-Zheng Zhan, Jing Xu, Chao-Ming Xia

**Affiliations:** ^1^Department of Parasitology, Medical College of Soochow University, Suzhou, China; ^2^Center for Genetic Epidemiology and Genomics, School of Public Health, Medical College of Soochow University, Suzhou, China; ^3^Department of Endocrinology, Second People’s Hospital of Hefei, Anhui, China; ^4^Department of Parasitology, Guangxi Medical University, Nanning, China

**Keywords:** liver fibrosis, miR-130a-3p, Ly6C^lo^, HSCs, schistosomiasis

## Abstract

Emerging evidences have highlighted the crucial role of microRNAs (miRNAs) in the liver cirrhosis, but the relationship between miR-130a-3p and liver cirrhosis is not entirely clear. As we all know, schistosomiasis, as one of the zoonoses, can lead to liver cirrhosis when it advances. In this study, we investigated the biological functions of miR-130a-3p on the liver fibrosis of schistosomiasis *in vivo* and *in vitro*. The mice infected with *Schistosoma japonicum* (*S. japonicum)* were treated with lentivirus vector (LV)-miR-130a-3p by hydrodynamic injection through the tail vein. Our findings showed significantly decreased expression of miR-130a-3p both in the serum of patients with cirrhosis and in the liver of mice infected with *S. japonicum*. The results showed that LV-miR-130a-3p could effectively enter into the liver and alleviate liver granulomatous inflammation and collagen deposition. Simultaneously, LV-miR-130a-3p-promoted macrophages presented the Ly6C^lo^ phenotype, concomitant with the decreased expression of the tissue inhibitor of metalloproteinases (TIMP) 1, and increased the expression of matrix metalloproteinase (MMP) 2, which contributed to the dissolution of collagen. Furthermore, overexpression of miR-130a-3p not only inhibited the activation and proliferation of hepatic stellate cells (HSCs) but also induced the apoptosis of HSCs. In addition, we also confirmed that miR-130a-3p enables to bind with mitogen-activated protein kinase (MAPK) 1 and transforming growth factor-beta receptors (TGFBR) 1 and TGFBR2 genes and inhibit the expressions of these genes. Our findings suggested that miR-130a-3p might represent as the potential candidate biomarker and therapeutic target for the prognosis identification and treatment of schistosomiasis liver fibrosis.

## Introduction

Schistosomiasis is one of the most serious parasitic diseases worldwide and is also raised as a critical public health issue affecting nearly 200 million people in tropical and subtropical regions (1). The main pathological characteristic of schistosomiasis is the massive deposition of eggs in the liver and intestine, which causes the release of soluble antigen and finally leads to granuloma and subsequent liver fibrosis (2, 3). Accordingly, liver fibrosis is an important risk factor for the development of ultimately cirrhosis, portal hypertension, and hepatocarcinoma (HCC), or even death (4). Since advanced liver fibrosis is generally regarded to be irreversible, there is no specific and effective antifibrotic program at the moment (5). Therefore, the development of an effective therapeutic target is urgent and necessary for the treatment or prevention of further progression of liver fibrosis. Up to date, a number of studies have implied the emerging roles of gene-based therapies; they might soon expand the therapeutic arsenal for several liver diseases (6).

MicroRNAs (miRNAs), made up of about 1–2% of all genes in mammals, are small, endogenous, noncoding RNAs containing about 22 to 26 nucleotides in length. It could regulate gene expression by the combination of translational repression and mRNA destabilization (6, 7). More than 60% of protein-coding genes are indicated to contain miRNA target sequences, and miRNAs have been implicated in a wide range of physiological as well as pathological processes. However, the deletion of many miRNAs genes usually did not appear in dominant phenotypes, suggesting that microRNAs exert only moderate effects on the levels of protein expression (8, 9). Previous studies have demonstrated that miRNAs play an important role in regulating various diseases, such as aging and cancer (10, 11). Accumulating evidences have demonstrated that miRNAs enable to regulate the activation of hepatic stellate cells (HSCs) and are involved in several types of chronic liver diseases, such as viral hepatitis, drug-induced liver injury, and autoimmune liver disease (8). Previous study has reported the potential roles of miR-130a-3p in diverse tumor cellular physiological process, including regulating cell growth, proliferation, apoptosis, metastasis, drug resistance, and tumor invasion. Furthermore, miR-130a-3p has also been shown to skew the polarization of macrophage to M2 and regulate profibrogenic gene expression in a chronic liver fibrosis model (12). However, the effects of miR-130a-3p on the activation, proliferation, and apoptosis of HSCs and the polarization of macrophages in the hepatic fibrosis progression of schistosomiasis are still unclear.

In this study, the expression of miR-130a-3p was decreased in the liver of *S. japonicum*-infected mice and in the serum of patients with cirrhosis. Then, the lentiviral vector-mediated miR-130a-3p (LV-miR-130a-3p) was injected into the mice infected with *S. japonicum* cercariae *via* the tail vein. After 8 weeks, the mice were tested for various indicators of liver fibrosis *in vivo.* We explored the role of LV-miR-130a-3p in the phenotype of macrophages *in vivo* and investigated the biological effects of miR-130a-3p on HSCs *in vitro*. Furthermore, the target genes regulated by miR-130a-3p were also predicted and verified. We found that miR-130a-3p could regulate the growth, proliferation, and apoptosis of HSCs and skew the polarization of macrophages to the Ly6C^lo^ phenotype, which might contribute to mitigating the pathogenesis of hepatic fibrosis. Our study could provide novel insights into the potential role of miR-130a-3p in the immunopathology of liver fibrosis in schistosomiasis.

## Materials and Methods

### Subjects and Ethics

Human serum samples were collected from the patients with cirrhosis caused by hepatitis B virus and healthy controls in Second People’s Hospital of Hefei (Hefei, China). Informed consent was obtained from all subjects. Animal experiments were carried out in strict accordance with the Regulations for the Administration of Affairs Concerning Experimental Animals (1988.11.1), and all efforts were made to minimize animal suffering. All animal procedures were approved by the Institutional Animal Care and Use Committee (IACUC) of Soochow University for the use of laboratory animals (Permit Number: 201604A136). The Institutional Ethical Committee of Soochow University and Second People’s Hospital of Hefei approved the study, and all experiments were conducted in accordance with the principles of the Declaration of Helsinki including any relevant details.

### Animal Model of Schistosomiasis

C57BL/6 mice (female, specific pathogen-free, 6–8 weeks of age, approximate weight of 18–20 g) were provided by the Experimental Animal Center of Soochow University (Suzhou, China). The mice were kept under specific pathogen-free (SPF) conditions with controlled temperature and humidity at the Laboratory Animal Research Facility of Soochow University (Suzhou, China). Oncomelania snails harboring *S. japonicum* cercariae were purchased from Shanghai Municipal Center for Disease Control and Prevention (Shanghai, China). To induce infection, all mice were exposed to the abdominal skin and challenged with 15 ± 1 *S. japonicum* cercariae.

All infected mice were randomly divided into three groups; each group (12 mice per group) was then injected with LV-miR-130a-3p, lentiviral vector-mediated negative control (LV-NC), or PBS, respectively, *via* the tail vein after cercariae challenge for 14 days. The concentration of LV-miR-130a-3p and LV-NC is 1 × 10^8^ TU, and the injection volume is 100 μl each one. Meanwhile, the mice in the PBS group were injected with 100 µl PBS.

### *In Vivo* Imaging System

After 72 h of LV-miR-130a-3p, LV-NC, or PBS injection, three mice were euthanized from the three groups; the tissue samples were harvested and placed into the *In-Vivo* Imaging System (IVIS Lumina XR III, PerkinElmer, Atlanta, GA).

### Histopathology and Fibrosis Measurement

The rest of the mice were sacrificed for later studies at 8 weeks post-infection. The left upper lobe of the liver specimens were harvested from the three groups and fixed in 10% neutral buffered formalin, embedded in paraffin blocks, cut into 4-μm-thick sections, and stained with hematoxylin–eosin (H&E) or Masson Trichrome for the measurement of the areas of the hepatic granulomas and evaluation of the degree of hepatic fibrosis. The prepared pathological sections, stained with H&E and MASSON staining, were photographed under bright-field images and hepatic egg granulomas (five per mouse) and the degree of hepatic fibrosis (five per mouse) images were measured with a computer image analysis system (Image-Pro Plus software,Media Cybernetics, Inc., Rockville, MD, USA).

The lower left lobe of the liver tissues weighed 100 mg accurately and was fully digested in 5% sodium hydroxide (NaOH) solution at 65°C for 1 h to count the *S. japonicum* eggs under bright field.

### Immunohistochemistry and Immunofluorescence Analysis

About 4-μm-thick paraffin sections were dewaxed and incubated according to the manufacturer’s instructions. The sections banded with α-smooth muscle actin (α-SMA) and collagen Type I (Col I) (Abcam Cambridge, MA) antibodies at 4°C overnight and then were incubated with the corresponding secondary antibodies for 45 min. The protein expressions of α-SMA and Col I were analyzed by the Image-Pro Plus software using the sum of the IOD (five per mouse).

### RNA Extraction and mRNA/miRNA Quantification

Total RNA of frozen liver tissues or cells was extracted using Trizol (Invitrogen, Carlsbad, CA, USA); human serum total RNA was harvested using a miRNeasy Serum/Plasma Kit (Qiagen, Germany). RNA concentrations were measured by Nanodrop 2000 (Thermo Fisher Scientific, MA, USA); 500 ng of RNA was reverse-transcribed into cDNA using the HiScript III 1st Strand cDNA Synthesis Kit (+gDNA wiper) (Vazyme, Nanjing, China). The mRNA level was analyzed by ABI QuantStudio 6 Flex (Thermo Fisher Scientific, MA, USA) using the ChamQ SYBR qPCR Master Mix (Low ROX Premixed; Vazyme, Nanjing, China) according to the manufacturers’ guidance. For miR-130a-3p detection, cDNA was synthesized by the TransScript^®^ miRNA First-Strand cDNA Synthesis SuperMix (Transgenbiotech, Beijing, China), and the relative expression level of miRNA was normalized to cel-miR-39 RNA. All data were analyzed using the threshold cycle (2−ΔΔ Ct). The primers are shown in **Table 1**. All experiments were repeated three times.

**Table 1:** The primers used for qRT-PCR.

Primer	Sense	Antisense
miR-130a-3p	CGGCAGTGCAATGTTAAAAGGGCAT	GATCGCCCTTCTACGTCGTAT
TGF-β1	CTGGATACCAACTACTGCTTCAG	TTGGTTGTAGAGGGCAAGGACCT
Col I	GCTCCTCTTAGGGGCCACT	CCACGTCTCACCATTGGGG
IL-4	TCTCGAATGTACCAGGAGCCATATC	AGCACCTTGGAAGCCCTACAGA
IL-13	CTTGCTTGCCTTGGTGGTCT	GCACAGGGGAGTCTGGTCTT
TIMP1	ACGGCATGGATCTCAAAGAC	GTGGGTGAGGAGCACGTAGT
iNOS	CACCTTGGAGTTCACCCAGT	ACCACTCGTACTTGGGATGC
MMP2	ACCTGAACACTTTCTATGGCTG	CTTCCGCATGGTCTCGATG
Arg-1	CTCCAAGCCAAAGTCCTTAGAG	AGGAGCTATCATTAGGGACATC
α-SMA	GGGAGCAGAACAGAGGAATG	CCAAACAAGGAGCAAAGACG
CCL2	AACTCTCACTGAAGCCAGCTCT	CGTTAACTGCATCTGGCTGA
CXCL2	ACCAACCACCAGGCTACA	TCAGGGTCAAGGCAAACT
CCL3	GATTCCACGCCAATTCATCG	AGGCATTCAGTTCCAGGTCA
CCL4	TTTCTCTTACACCTCCCGGC	AGCTGCTCAGTTCAACTCCA
MAPK1	TCTCCTCTGTGTTGTCCTCCTTCC	GGCTGCCGCTCGACTTATGC
TGFBR1	CATTGCTGGTCCAGTCTGCTTCG	TGGTGAATGACAGTGCGGTTATGG
TGFBR2	ATCTGTGAGAAGCCGCATGAAGTC	AGAGTGAAGCCGTGGTAGGTGAG
cel-miR-39 5’	UCACCGGGUGUAAAUCAGCUUG	GATCGCCCTTCTACGTCGTAT
GAPDH	CAACTTTGGCATTGTGGAAGG	ACACATTGGGGGTAGGAACAC

TGF-β1, transforming growth factor beta 1; Col I, type I collagen; iNOS, inducible nitric synthase; IL, interleukin; MMP2, matrix metalloproteinase 2; Col I, type 1 collagen; Arg-1, arginase-1; α-SMA, α-smooth muscle actin; CCL, chemokine (C-C motif) ligand; CXCL, chemokine (C-X-C motif) ligand; GAPDH, glyceraldehyde-3-phosphate dehydrogenase.

### HSC Isolation, Identification

Four mice from each group (*S. japonicum* infected mice and non-infected mice) were sacrificed.

Firstly, RPMI 1640 (3.5 ml/min) was perfused from the hepatic portal vein of the mice and then 0.04% Collagenase I in RPMI 1640 (3.5 ml/min). The perfused liver tissue was collected to ground and continuously digested by type IV collagenase for 35 min. The nonparenchymal cells were collected and the HSCs were purified by density gradient centrifugation with Optiprep. HSCs were fixed using 4% paraformaldehyde for 60 min; 10% normal goat serum was used to inhibit unspecific binding. GFAP staining was done for 12 h at 4°C using rabbit anti-mouse GFAP polyclonal antibody. Secondary antibody incubation was followed by biotin-labeled goat anti-rabbit IgG for 30 min at 37°C and further incubation with SABC-Cy3 for 30 min at 37°C. Finally, slides were mounted using a Vectashield Mounting medium (Millipore). Each slide was captured using a microscope (OLYMPUS), and the ratio of GFAP+ cell is calculated.

### Cell Culture and Transfection

JS1, a well-characterized mouse HSC cell line, was purchased from Beinna Biology Institute. JS1 cells were cultured in DMEM high glucose (Hyclone) supplemented with 10% fetal bovine serum, 4 mmol/L L-glutamine, and 100 IU/mL penicillin/streptomycin maintained in a 5% CO_2_ incubator at 37°C. To enhance/inhibit the miR-130a-3p expression, miR-130a-3p Agomir or miR-130a-3p Antagomir was transfected into JS1 cells. The transfection of JS1 cells was classified into Agomir-130a-3p, Agomir NC, Antagomir-130a-3p, and Antagomir NC groups. The synthetic miR-UP Agomir and Agomir NC, and miR-Down Antagomir and Antagomir NC were acquired from Shanghai GenePharma (Shanghai, China). The transfection was implemented using the Lipofectamine 3000 (Invitrogen, CA, USA) kit according to the manufacturer’s instructions. Before the transfection process, 5 μl RNA duplexes (100 pmol) and 5 μl Lipofectamine 3000 were respectively diluted using 125 ml Opti-MEM medium (Gibco, CA, USA). The mixture was left to stand for 10 min at room temperature, and then it was added into the corresponding cell culture wells, which were put back into the cell incubator for further incubation. After 6 h, the medium was completely replaced with a fresh medium. Then, the cells continued to be incubated for another 48 to 72 h for subsequent experiments.

### Liver Macrophages Isolation

Firstly, Hanks’ balanced salt solution (3.5 ml/min) was perfused from the hepatic portal vein of the mice infected with *S. japonicum* cercariae after 8 weeks. Then, 20 ml 0.05% type IV collagenase in Hanks’ balanced salt solution were used to perfuse the liver tissue (3.5 ml/min). The perfused liver tissue was collected to ground and continuously digested by type IV collagenase for 35 min. Digested cell suspension was filtered through sterile nylon gauze (50 nm), removing as much connective tissue and parenchymal cells as possible. The cell suspension was added to two-step Percoll separation (GE Healthcare Life Sciences, PA, USA), and macrophages were acquired from the layers between the 50% and the 25% Percoll separation.

### Flow Cytometry (FCM) Analysis

The isolated liver macrophages were further washed twice by DMEM containing 1% FBS (Gibco, Grand Island, NY, USA), removing the supernatant, and then resuspended the cells with PBS; final cell concentration was around 1 × 10^6^ cells/100 μl PBS. The cells were stained with FITC-labeled antimouse CD11b (Biogems, CA, USA), PE-labeled antimouse F4/80 (Biolegend, CA, USA), and PE-cy7-labeled antimouse Ly-6C (BD Bioscience, CA, USA) antibodies for incubation at 4°C for 30 min, and then washed twice in PBS and detected by FCM (BD FACSVerse system, CA, USA). The apoptosis of JS1 was detected by the PE Annexin V Apoptosis Detection Kit (BD Biosciences, CA, USA) based on the manufacturer’s instructions.

### Western Blotting

Liver tissue or JS1 cells were fully lysed with lysis buffer (BestBio, Shanghai, China) at 4°C, total proteins were extracted from lysate suspension, and their concentrations were measured using a BCA protein assay kit. Each protein sample that makes a concentration of 20 μg was electrophoretically separated on 8/15% polyacrylamide gels and then transferred into PVDF membranes (Millipore, Burlington, MA, USA). The membranes were blocked and bound with 5% skim milk for 2 h at room temperature, and probed with antibodies to Col I (1:800) (Bioss, Beijing, China), α-SMA (1:1,000) (Abcam Cambridge, MA), ERK1/2 (1:1,000) (Abcam Cambridge, MA), TGFRB1 (1:1,000) (Proteintech, Chicago, USA), TGFRB2 (1:1,000) (Proteintech, Chicago, USA), and GAPDH (1:2,000) (Abcam Cambridge, MA) at 4°C overnight, and the corresponding HRP-conjugated secondary antibody (Bioss, Beijing, China) was bound for 45 min at room temperature. Then, antigen-antibody reaction was detected by an enhanced chemiluminescence assay (ECL) kit (Millipore, MA, USA). The final results were evaluated with the Image J software, which semiquantitatively estimated the intensity of the grayscale images.

### Target Gene Prediction

The miR-130a-3p was subjected to five of the most used online RNA databases to predict the target genes regulated by miR-130a-3p, including miRDB (http://mirdb.org/), mirTar (http://mirtar.mbc.nctu.edu.tw/human/index.php), mirDIP (http://ophid.utoronto.ca/mirDIP/index_confirm.jsp), TargetScan (http://www.targetscan.org/vert_72/), and miRWalk (http://mirwalk.umm.uni-heidelberg.de/human/mirna/191/).

The overlapping genes among the five databases were obtained using the Vern diagram. An online gene set enrichment (GSE) analysis tool (KOBAS) (http://kobas.cbi.pku.edu.cn/) was applied to evaluate the functional annotation and enrichment among the overlapped genes. Three target genes of MAPK1, TGFBR1, and TGFBR2 were selected for further experimental verification.

### Dual Luciferase Reporter Assay

To confirm if MAPK1, TGFBR1, and TGFBR2 are the target genes of miR-130a-3p, the synthetic 3’ UTR fragments of MAPK1, TGFBR1, and TGFBR2 genes were inserted into the 3’ UTR of the miRNA expression reporter vector (pmirGLO) (Promega, WI, USA) to construct MAPK1-WT, TGFBR1-WT, and TGFBR2-WT vectors, respectively. The vectors of MAPK1-MUT, TGFBR1-MUT, and TGFBR2-MUT were also constructed by inserting the mutant 3’ UTR fragments of MAPK1, TGFBR1, and TGFBR2 genes into the 3’ UTR of pmirGLO, respectively. The luciferase reporter plasmids containing MAPK1-WT/MAPK-MUT, TGFBR1-WT/TGFBR1-MUT, and TGFBR2-WT/TGFBR2-MUT were then cotransfected with Agomir-130a-3p into HEK293T. After transfection for 48 h, the cells were collected and lysed. The activity of luciferase was measured by a TransDetect^®^ Double-Luciferase Reporter Assay Kit (Transgen, Beijing, China) and a SpectraMax iD3 multimode microplate reader (San Jose, CA, USA). Renilla luciferase was used as an internal reference. The experiment was repeated three times.

### Statistical Analysis

Data were presented as mean ± standard deviation (SD). Comparison between two groups was evaluated by Student’s t-test. Comparisons of data of more than two groups were implemented by using one-way analysis of variance (ANOVA) or nonparametric analysis (Kruskal–Wallis test). Statistical analysis was performed with the use of the Statistical Package for the Social Sciences (SPSS) statistical software, version 23.0 (SPSS Inc., Chicago, IL, USA). The level of statistical significance was set at two-tailed *p*-value < 0.05.

## Results

### The Levels of miR-130a-3p Are Decreased in the Liver of Mouse Schistosomiasis and in the Serum of Cirrhosis Patients

**Figure 1:**
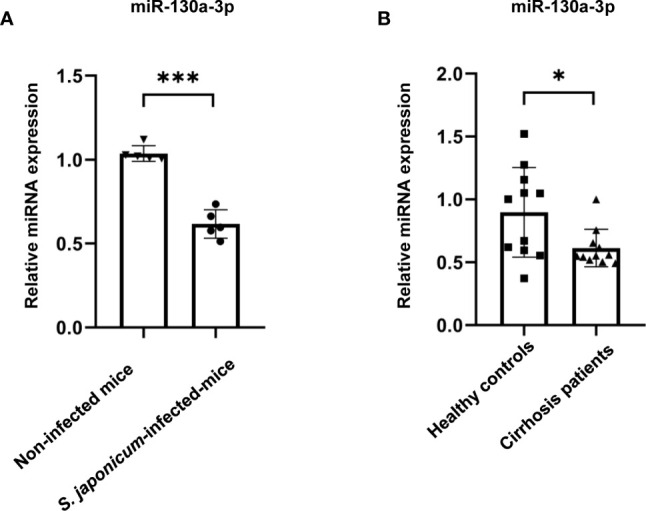
Validation of the levels of miR-130a-3p in the livers of fibrotic mice and in the serum of fibrotic patients. The levels of miR-130a-3p in the liver of fibrotic mice and in the serum of cirrhosis patients were analyzed by quantitative real-time PCR (qRT-PCR). The expression of miR-130a-3p in *S. japonicum*-infected mice liver **(A)** and in serum of cirrhosis patients **(B)**. *p < 0.01 and ***p < 0.001 (n = 4 each group).

To investigate whether there were any differences of miR-130a-3p levels in the liver between mice infected with *S. japonicum* and non-infected mice, initially, we established the liver fibrosis of schistosomiasis in a murine model (**Supplementary Figure 1**) and measured the levels of miR-130a-3p in the liver; the results showed that there were significantly decreased levels of miR-130a-3p in the liver of mice infected with *S. japonicum* after 8 weeks as compared to the non-infected liver (**Figure 1A**). Simultaneously, we also compared the serum expression of miR-130a-3p in patients with cirrhosis and healthy controls and found significantly lower serum miR-130a-3p levels in cirrhosis patients as compared to controls (**Figure 1B**). These findings provided the strong indication of the possible links between miR-130a-3p and the development of liver fibrosis.

### LV-miR-130a-3p Attenuates the Pathological Progression in Mouse Schistosomiasis

**Figure 2:**
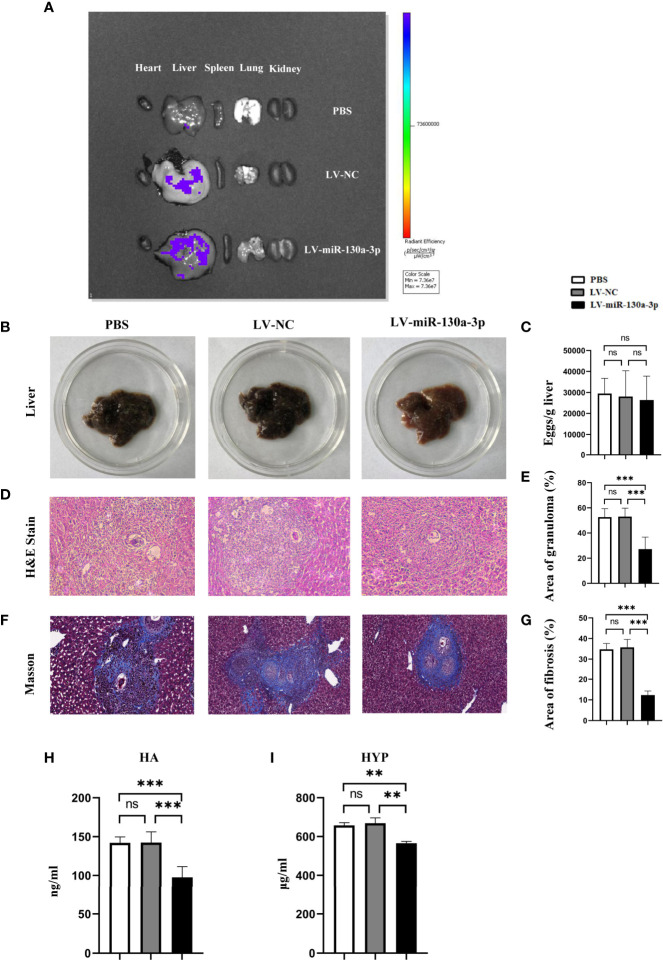
LV-miR-130a-3p alleviated the pathological progression of liver fibrosis in *S. japonicum* infection. The mice were infected with 14 ± 1 cercariae of *S. japonicum* through abdominal skin in different groups. We injected LV-miR130a-3p, LV-NC, or PBS into the mice through the tail vein. After 72 h, the distributions of green fluorescent protein (GFP) in the heart, liver, spleen, lung, and kidney were detected by *In Vivo Imaging System* **(A)**. After 8 weeks, the serum and the liver of different groups were collected. The photos of mice livers were captured **(B)**. Eggs in the liver were counted **(C)**. Liver tissue section was stained with H&E and the original magnification of stained liver sections was 100×. The granulomas were showed with quantitative analysis **(D, E)**. Liver tissue section was stained with Masson, and the original magnification of stained liver sections was 100×. The fibrosis was showed with quantitative analysis **(F, G)**. Sera HA was assayed by enzyme-linked immunosorbent assay **(H)**. Liver HYP was checked by the alkaline lysis method **(I)**. Data represent mean ± SD from multigroup experiments. **p < 0.01 and ***p < 0.001 (n = 4 each group). ns, no significance.

Initially, we used the *In Vivo* Imaging System to explore the distribution of LV-miR-130a-3p in the major organs of mice after 72 h of tail intravenous infusion. The results found that the distributions of the green fluorescent protein (GFP) expressed by LV-miR-130a-3p and LV-NC were mainly located in the liver, suggesting that miR-130a-3p could colonize in the liver *via* the lentivirus vector through the tail vein injection (**Figure 2A**). Moreover, we found that the GFP expressed by LV-miR-130a-3p was located in HSCs by a fluorescence microscope, suggesting that hydrodynamic injection of LV-miR-130a-3p could deliver miR-130a-3p to HSCs (**Supplementary Figure 2**).

We speculated that miR-130a-3p may exert as an anti-inflammatory subset in suppressing the response of egg-induced granulomatous and alleviating liver fibrosis in mice infected with *S. japonicum*. To test this hypothesis, we respectively treated mice with LV-miR-130a-3p, LV-NC, and PBS using the tail vein injection after being infected with *S. japonicum* for 2 weeks. At the time mice were infected with *S. japonicum* for 8 weeks, liver tissue samples were obtained for biochemistry test and pathological evaluation. The liver sample of LV-miR-130a-3p group appeared to be less symptomatic, showed a fleshy pink appearance, and had just fewer nodules on the surface (**Figure 2B**). However, there was no obvious difference with respect to the egg burden between LV-miR-130a-3p and LV-NC groups (**Figure 2C**). H&E and Masson staining of the liver section showed that, as compared to the control group, the areas of hepatic granulomas and the content of hepatic fibrosis were significantly reduced in the LV-miR-130a-3p group (**Figures 2D–G**). In addition, the serum levels of hyaluronic acid (HA) in sera (**Figure 2H**) and the expression of hydroxyproline (HYP) in the liver (**Figure 2I**) were significantly decreased in the LV-miR-130a-3p group than those in the LV-NC group. These results demonstrated that LV-miR-130a-3p enables to colonize in the liver through the tail vein injection and ameliorates the liver pathology of mice infected with *S. japonicum*.

### LV-miR-130a-3p Decreases Col I and α-SMA Expression

**Figure 3:**
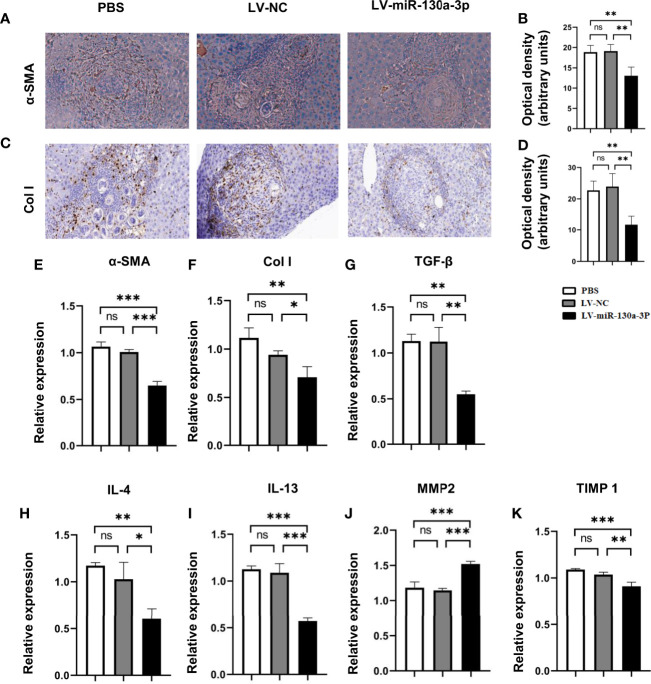
LV-miR-130a-3p decreased the levels of Col I by reduced expression of proinflammatory factor. The α-SMA protein level in the liver was evaluated by IHC with quantitative analysis of optical density **(A, B)**. The Col I protein level in the liver was evaluated by IHC with quantitative analysis of optical density **(C, D)**. Liver RNA was extracted and the expressions of α-SMA and Col I were analyzed by qRT-PCR **(E, F)**. Liver RNA was extracted and the expressions of proinflammatory factor, TGF-β, IL-4, IL-13, MMP2, and TIMP1were analyzed by qRT-PCR **(G–K)**. Data represent mean ± SD from multigroup experiments. *p < 0.05, **p < 0.01 and ***p < 0.001 (n = 4 each group). ns, no significance.

To elucidate the mechanism of LV-miR-130a-3p alleviating liver pathology in schistosomiasis, immunohistochemical (IHC) staining and qRT-PCR were used to detect the protein and mRNA expressions of α-SMA and Col I in LV-miR-130a-3p and LV-NC treated liver of mice infected with *S. japonicum*. The results indicated that both the protein and mRNA expressions of α-SMA and Col I showed significant reduction in the LV-miR130a-3p group as compared to the LV-NC group (**Figures 3A–F**).

Simultaneously, after LV-miR130a-3p treatments, the mRNA levels of pro-fibrosis factors transforming growth factor (TGF)-β1, interleukin (IL)-4, and tissue inhibitor of metalloproteinases (TIMP) 1 in HSCs were lower than those in the LV-NC treatment, but the anti-fibrosis factor of matrix metalloproteinase (MMP) 2 showed an increase in the LV-miR-130a-3p group than that of the LV-NC group (**Figures 3G–K**). Thus, we concluded that LV-miR-130a-3p could reduce the deposition of Col I through producing less α-SMA, TGF-β1, IL-4, and TIMP1 and more MMP2, which are responsible for the deactivation of HSCs and dissolve collagen in liver pathology of mice infected with *S. japonicum*, respectively.

### LV-miR-130a-3p Upregulate the Expression of Ly6C^lo^ Macrophages

**Figure 4:**
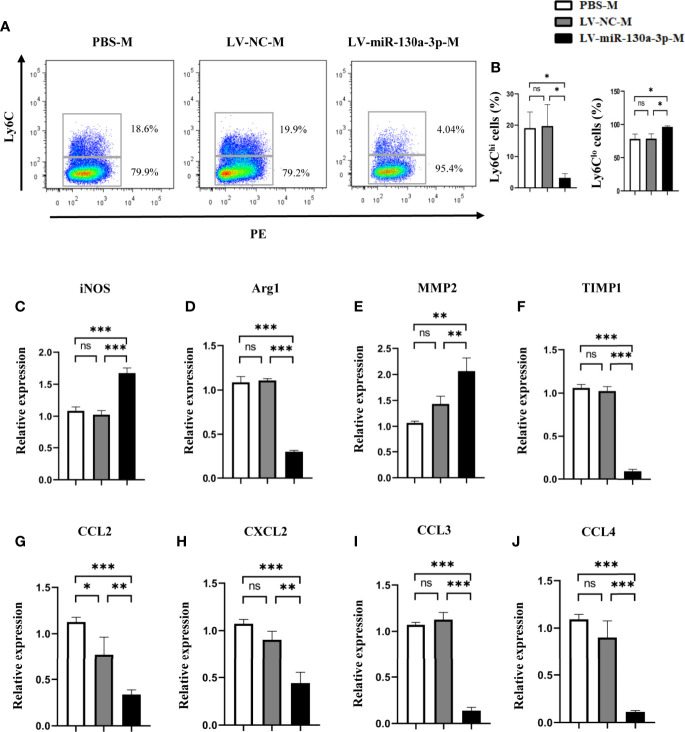
LV-miR130a-3p drove liver macrophages to Ly6C^lo^ skewing. Macrophages were isolated from the liver in *S. japonicum*-infected mice from different groups by density gradient centrifugation and then were purified according to the surface marker of F4/80 and CD11b by FCM. The expressions of Ly6C on the LV-miR130a-3p-M surface were respectively detected by FCM **(A, B)**. The liver macrophage RNA was extracted to analyze inflammatory chemokines **(C-F)**. The liver macrophage RNA was extracted to analyze chemokines, CCL2, CXCL2, CCL3, and CCL4 **(G–J)**. Data represent mean ± SD from multigroup experiments. *p < 0.05, **p < 0.01 and ***p < 0.001 (n = 4 each group). ns, no significance.

In the chronic inflammation model, miR-130a-3p could degrade the macrophage profibrogenic gene M2 expression. Given the prior evidence, we hypothesized that miR-130a-3p could also regulate the expression of proinflammatory factor Ly6C^hi^ in the macrophage, which is involved in the progression of schistosomiasis liver fibrosis. To test our hypothesis, we isolated the liver macrophage from different treatment groups to identify the expression of Ly-6C. As expected, the LV-miR-130a-3p group showed decreases in the numbers of Ly6C^hi^ macrophages but increases in Ly6C^lo^ macrophages detected by FCM (**Figures 4A, B**). Furthermore, we harvested liver macrophage from different groups for measurements of the mRNA expressions of inducible nitric synthase (iNOS), Arginase (Arg)1, MMP2, and TIMP1 by qRT-PCR. The results suggested that, compared to the LV-NC group, the mRNA levels of MMP2 and iNOS were markedly elevated in the LV-miR-130a-3p group, but the mRNA levels of Arg1 and TIMP1 decreased in the LV-miR-130a-3p group (**Figures 4C–F**). To evaluate the recruitment function of LV-miR130a-3p on monocytes, the mRNA levels of several chemokines in the liver samples from different groups were measured by qRT-PCR. It is surprising that the mRNA expression levels of chemokine (C-C motif) ligand (CCL)2, chemokine (C-X-C motif) ligand (CXCL)2, CCL3, and CCL4 were lower in the liver of the LV-miR-130a-3p group than those in the LV-NC group (**Figures 4G–J**). Taken together, our data revealed that miR-130a-3p could also promote macrophages skew to Ly6C^lo^ and decrease the ability to recruit monocytes into the liver to convert Ly6C^hi^ macrophages, indirectly relieving the liver pathology of mice infected with *S. japonicum.*

### The Regulating Effects of miR-130a-3p on the Activation and Collagen Deposition of JS1 Cells

**Figure 5:**
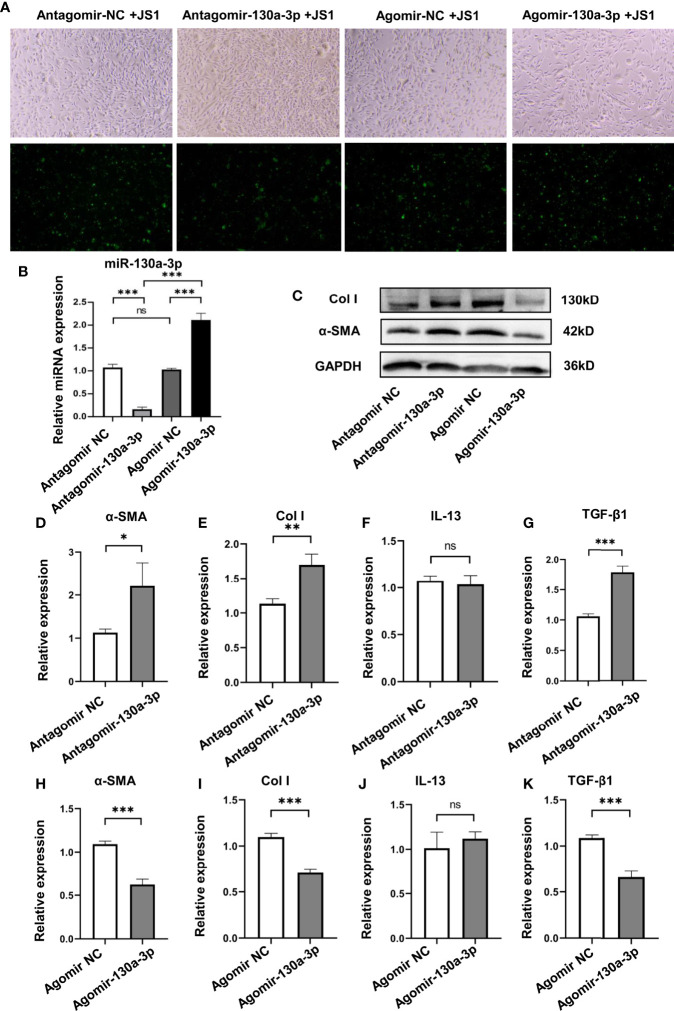
Overexpression of miR130a-3p reduces the activation of JS1 and the expression of fibrogenic factors. JS1 cells were transfected with Agomir-130a-3p/Agomir NC or Antagomir-130a-3p/Antagomir NC **(A)**. The transfected JS1 cells were collected after 72 h; the expression of miR-130a-3p was validated by qRT-PCR **(B)**. The transfected JS1 were collected after 72 h to assay the protein level of α-SMA and Col I by WB **(C)**, and the mRNA levels of α-SMA, Col I, IL-13, and TGF-β1 in the gene level were analyzed by qRT-PCR **(D–K)**. Data represent mean ± SD from multigroup experiments. *p < 0.05, **p < 0.01 and ***p < 0.001 (n = 4 each group). ns, no significance.

To elucidate the mechanism of miR-130a-3p in alleviating liver pathology in schistosomiasis, we investigated the effect of miR-130a-3p on the activation and collagen deposition of JS1 cells, which represented a well-characterized mice HSC cell line. The chemically modified miRNA mimics (Agomir-130a-3p/Agomir NC) and miRNA inhibitors (Antagomir-130a-3p/Antagomir NC) conjunct with GFP were transfected into JS1 cells. The transfection efficiency was evaluated by visual observation of the distribution of GFP using a fluorescence microscope, and the level of miR-130a-3p was detected by qRT-PCR. As compared to nontransfection cells, there were a large number of green fluorescence in JS1 cells that could be observed after transfection of Agomir-130a-3p/Agomir NC or Antagomir-130a-3p/Antagomir NC, indicating that the transfection was effective (**Figure 5A** and **Supplementary Figure 3**). Furthermore, the levels of miR-130a-3p in JS1 cells showed a comparable increase after transfection of Agomir-130a-3p compared with Agomir NC, and there was also a decrease in miR-130a-3p in the Antagomir-130a-3p group compared to the Antagomir NC group (**Figure 5B**). We used WB and qRT-PCR to determine whether miR-130a-3p could successfully regulate the expression of α-SMA and Col I in JS1 cells at levels of mRNA and protein. Consistent with previous *in vivo* findings, the results showed that transfection with Agomir-130a-3p could obviously decrease the expression of α-SMA and Col I on both the mRNA and the protein levels in JS1 cells in comparison with Agomir NC (**Figures 5C, H, I**). When transfected with Antagomir-130a-3p in JS1 cells, there was a significant increase in the expression of α-SMA and Col I on both the mRNA and protein levels than when transfected with Antagomir NC (**Figures 5C, D, E**). The protein expressions of α-SMA and Col I were quantified and normalized in **Supplementary Figure 4**.

In addition, we also explored if the transfection of Agomir-130a-3p and Antagomir-130a-3p could influence the levels of IL-13 and TGF-β1 on JS1 cells; we found Agomir-130a-3p decreased the expression of TGF-β1, but Antagomir-130a-3p has effects on the increase in TGF-β1. On the other hand, there were no significant changes in IL-13 concentrations in both Agomir-130a-3p and Antagomir-130a-3p groups (**Figures 5F, G, J, K**).

### miR-130a-3p Inhibits the Proliferation and Promotes Apoptosis of JS1 Cells *In Vitro*

**Figure 6:**
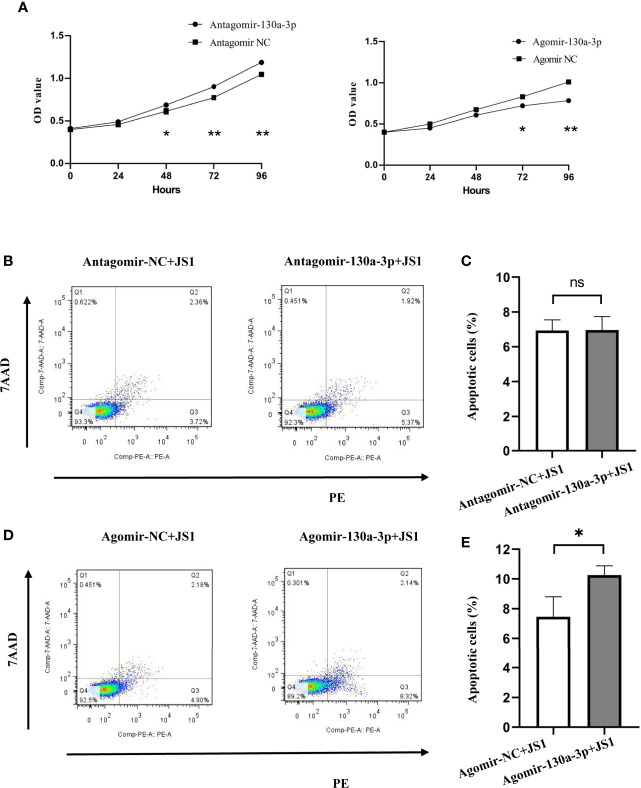
Overexpression of miR-130a-3p inhibited the proliferation and growth, and induced apoptosis of JS1. The proliferation of transfected JS1 cells was tested at 0, 24, 48, 72, and 96 h by CCK8 assay **(A)**. The transfected JS1 cells were collected after 72 h; cell apoptosis was assessed by FCM **(B–E)**. Data represent mean ± SD from multigroup experiments. *p < 0.5 (n = 4 each group). **p < 0.01; ns, no significance.

As shown in **Figure 6A**, the proliferation of JS1 cells suggested that the ability of proliferation was significantly decreased in the Agomir-130a-3p group compared with the Ago-NC group; however, the results were reversed in Antagomir-130a-3p and Antagomir-NC. Simultaneously, the influence of miR-130a-3p on the apoptosis of JS1 was investigated by FCM. We found that there was a significantly increased cell apoptosis rate in the Agomir-130a-3p group compared with the Agomir NC group. Nevertheless, there were no significantly differences regarding the cell apoptosis rate between Antagomir-130a-3p and Antagomir-NC groups (**Figures 6B–E**). These evidences revealed that the overexpression of miR-130a-3p could repress proliferation and induce apoptosis of JS1.

### The Regulating Effects of miR-130a-3p Targeting Genes of MAPK1, TGFBR1, and TGFBR2

**Figure 7:**
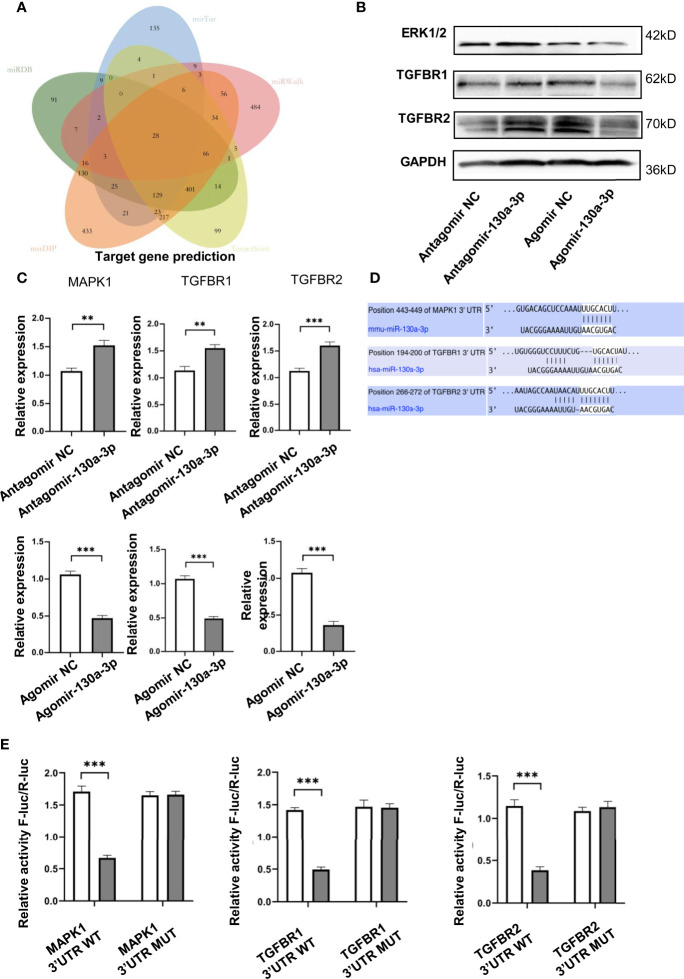
miR-130a-3p target genes prediction. Venn diagram showing the overlap of the miR-130a-3p regulated target genes in the following five algorithms: TargetScan, mirDIP, miRDB, mirTar, and mirDIP **(A)**. JS1 cells were transfected with the Agomir-130a-3p/Agomir NC, Antagomir-130a-3p/Antagomir NC for 72 h; the protein and mRNA expression of ERK1/2, TGFBR1, and TGFBR2 were detected by WB and qRT-PCR, respectively **(B, C)**. The binding sites of miR-130a-3p in 3′ UTR of MAPK1, TGFBR1, and TGFBR2 were predicted **(D)**. The wild-type and mutants 3’ UTR of MAPK1, TGFBR1, and TGFBR2 were cloned into pmirGLO, dual-luciferase reporter assay performed on 293 T cells transfected with MAPK1, TGFBR1, and TGFBR2 UTR reporter plasmid together with Agomir-130a-3p or Agomir N **(E)**. **p < 0.01 and ***p < 0.001 (n = 4 each group).

To further verify the genetic molecular mechanisms of miR-130a-3p in liver fibrosis, the potential target genes regulated by miR-130a-3p were identified. Five online RNA databases (miRDB, mirTar, mirDIP, TargetScan, and miRWalk) were applied to predict the potential target genes regulated by miR-130a-3p. The visual depicted Venn diagram showed that there are 28 potential target genes that overlap among the five databases (**Figure 7A**). To further verify the possible target genes regulated by miR-130a-3p, we used the online KOBAS tool to evaluate the functional annotation and enrichment among the overlapped genes. Three target genes of MAPK1, TGFBR1, and TGFBR2 were selected for further test using qRT-PCR and WB. The findings revealed that both the mRNA and protein expressions of MAPK1, TGFBR1, and TGFBR2 were downregulated in the Agomir-130a-3p group as compared to the Agomir NC group. On the other hand, in comparison to the Antagomir NC group, the Antagomir-130a-3p group had increased mRNA levels of MAPK1, TGFBR1, and TGFBR2 but showed similar protein expressions (**Figures 7B, C**). The protein expressions of ERK1/2, TGFBR1, and TGFBR2 were quantified and normalized in **Supplementary Figure 5**. In addition, the mRNA levels of MAPK1, TGFBR1, and TGFBR2 in the liver tissue were decreased in the LV-miR-130a-3p group compared to those in the LV-NC or PBS groups (**Supplementary Figure 6**). Moreover, as shown in **Figure 7D**, miR-130a-3p had binding cites in the 3’ UTR of MAPK1, TGFBR1, and TGFBR2.

To verify if MAPK1, TGFBR1, and TGFBR2 are the direct target of miR-130a-3p, the dual luciferase reporter plasmids containing the 3’ UTR of MAPK1, TGFBR1, and TGFBR2 flanking the putative miR-130a-3p binding sites were constructed, respectively. The dual luciferase reporter assay showed that miR-130a-3p enables to significantly reduce the luciferase activity of WT-MAPK1, WT-TGFBR1, and TGFBR2 compared to MUT-MAPK1, MUT-TGFBR1, and MUT-TGFBR2 (**Figure 7E**). These findings suggested that miR-130a-3p could bind with MAPK1, TGFBR1, and TGFBR2 genes and inhibit the expression of these genes, which might contribute to the alleviation in liver fibrosis.

## Discussion

Up to date, accumulating evidences have suggested that miRNAs play important roles and might be regarded as a potential therapeutic target in regulating the progression and development of several liver diseases (13–16). Previous reports have demonstrated that there were increased levels of miR-130a-3p in myocardial injury, and downregulation of miR-130a-3p could remarkably relieve hypoxia-induced inflammation and inhibit the expression of fibrosis-related protein (17). However, it has also been found that there were decreased levels of miR-130a-3p in both nonalcoholic steatohepatitis (NASH) patients and a high fat diet-induced mouse model, and the overexpression of miR-130a-3p could rescue the severity of NASH fibrosis in the mouse model (18). Regarding the two literature reports that miR-130a-3p has opposite effects on fibrosis-related protein, we believe that the possible reason is that different models of disease cause different effects.

As we all know, once the liver is continuously infected, either by viral or schistosomiasis, it will eventually develop to liver fibrosis and cirrhosis. In this study, we used the mice infected with *S. japonicum* and patients with cirrhosis to investigate the link between miR-130a-3p and liver fibrosis. The results showed that the levels of miR-130a-3p were significantly decreased in the liver of mice infected with *S. japonicum* as compared to non-infected mice. Furthermore, the serum levels of miR-130a-3p were also decreased in patients with cirrhosis than those in healthy subjects. These findings indicated that the reduced miR-130a-3p might have an association with liver fibrosis.

LV, as a dominant vector, enables to continuously express the target gene for a long time. It has been demonstrated that hydrodynamic tail-vein injections could effectively transport LV into the liver with the highest concentrations (19). In addition, Brown et al. suggested that LV is able to enter into liver nonparenchymal cells more efficiently than hepatocytes (20). Granuloma reactions, occurring approximately 5–6 weeks after schistosomiasis infection, are mainly composed of a mass of nonparenchymal cells, including macrophages, lymphocytes, eosinophils, mast cells, and mononuclear cells. The accumulation of nonparenchymal cells around the schistosome eggs exerts an immunological regulated effect to protect the host from inflammatory response caused by an excessive secretion of soluble antigens (21, 22). Thus, in our study, the mice model of schistosomiasis treated with LV-miR-130a-3p or LV-NC or PBS by tail vein injection was built. We found reduced areas of hepatic granuloma and the decreased content of liver fibrosis in the LV-miR-130a-3p treated group as compared to those of LV-NC and PBS groups. However, the number of schistosome eggs in these groups showed no significant differences. Thus, we may speculate that the overexpression of miR-130a-3p might contribute to alleviate liver granulomas and fibrosis in mice infected with *S. japonicum* through regulating the immune response.

Macrophages, as one of the important immune effector cells, are made up of about 30% of granuloma cells (23). In general, macrophages are classified into classically activated macrophage (M1) and alternatively activated macrophage (M2) (24). Literatures have implied that miRNAs could affect the maturation of monocytes and regulate immune inflammatory response through skewing the polarization of macrophages. Previous studies indicated that miRNAs tightly control the macrophage polarization continuum from proinflammatory M1 to pro-reparative M2 by regulating the expression of key transcription factors, which are involved in the regulation of macrophage polarization (25–27). Su et al. unveiled that downregulated miR-130a-3p levels were associated with the profibrogenic macrophages program through stimulating Th2 and maintaining M2 polarization (12). Despite the classification of traditional macrophages of M1 and M2 phenotype macrophages, researchers have identified that macrophages can also be divided into Ly6C^hi^ and Ly6C^lo^ mononuclear cells, according to the expression of Ly6C. Ly6C^hi^ macrophages play an important role in proinflammatory at the early stage of liver fibrosis and show the ability to recruit the circulating monocytes (28). The infiltration of Ly6C^hi^ monocytes into the liver relied on the interaction of numerous chemokines and their receptors, such as Ccl2-Ccr2, Ccl1-Ccr8, and Ccl3/4/5-Ccr1/5. However, Ly6C^hi^ macrophages convert to Ly6C^lo^ macrophages in the late stage to restore scar tissues. To the best of our knowledge, there was no study that was conducted to explore the effect of miR-130a-3p on Ly6C-expressing macrophages. In this study, we discovered that miR-130a-3p had an ability to upregulate the expression of Ly6C^lo^ macrophages, which was displayed as a phenotype of restorative macrophages and suppressed the expression of the profibrotic genes of Arg1 and TIMP1, but increased the expression of MMP2 and iNOS, thus minimizing granuloma formation and alleviating the fibrotic process.

Different from other liver fibrosis caused by carbon tetrachloride (CCL4) and bile duct ligation (BDL), the activated HSCs, not the necrosis of the hepatocyte, are a crucial component of the process of schistosomiasis liver fibrosis (29–32). HSCs, as nonparenchymal cells, are responsible for the storage and metabolism of vitamin A in a quiescent state. There are numbers of risk factors, such as chronic hepatitis virus, *S. japonicum*-infection and alcohol consumptions, that could activate quiescent HSCs into activated HSCs, which then transform into proliferative, fibrogenic, proinflammatory, and contractile myofibroblasts, and produce α‐SMA and collagen, further contributing to the development of liver fibrosis (33, 34). Accumulating evidences have demonstrated that inhibition of liver fibrosis mainly relies on inducing apoptosis in activated HSCs (35). Therefore, a better understanding of the mechanisms of HSC activation, proliferation, and apoptosis would be very helpful for the clinical treatment of liver fibrosis. Our study found that the miR-130a-3p could effectively promote the apoptosis of HSCs and inhibit the activation and proliferation of HSCs, and alleviate the content of hepatic fibrosis. In summary, all of these results suggested that miR-130a-3p may be a promising target against the hepatic fibrosis of schistosomiasis.

To investigate miR-130a-3p regulated target genes, the potential target genes of miR-130a-3p were predicted in five RNA databases. There were a total of 28 overlapped target genes. Among the 28 target genes, we considered that MAPK1, TGFBR1, and TGFBR2 represent the potential target genes that could be regulated by miR-130a-3p and involved in the liver fibrosis of schistosomiasis. MAPK signaling is mainly composed of extracellular signal-regulated kinase, protein-regulated kinase P38, and C-Jun N-terminal kinase (JNK) (36). It is known that the MAPK signaling pathway is involved in the process of liver fibrosis by regulating HSCs, including directly affecting the activation and proliferation of HSCs (37), indirectly affecting secreted intracellular and extracellular cytokines (such as TGF-β and TNF-α) on the influence of HSC activation (38), or indirectly affecting the activation of HSCs by the activation of the JNK pathway in the recovery stage of liver fibrosis (39). It is well known that iNOS-nitric oxide (NO) plays an important role in the phenotypic changes of macrophages; M1 could always be activated by cytokines of IFN-γ and TNF-α, which are primarily secreted by Th1 cells, and produces iNOS to kill pathogen through generating NO. Meanwhile, M2 is mainly induced by Th2 cell factors of IL-4 and IL-13 and produces Arg-1 to competitively use l-arginine to synthesis L-ornithine, which is effectively translated into proline and polyamine and reduces the generation of NO (40). Previous studies have also reported that MAPKs are important regulators of iNOS-NO (41). The activation of MAPK could modulate the p38 and JNK pathway resulting in LPS-induced iNOS expression in RAW 264.7 macrophages (42, 43). Our results showed that the expression of MAPK1 was reduced in the transfected cells with Agomir-130a-3p both at the mRNA and protein levels. It suggested that the increase in miR-130a-3p could inhibit MAPK1 expression, promote the apoptosis of HSCs, and skew the polarization of macrophages towards Ly6C^lo^, thus rescuing the development of liver fibrosis.

The TGF-β superfamily has also been indicated to play a predominant role in the development of liver injury and damage. TGF-β not only promotes myofibroblast transdifferentiation and matrix synthesis but also activates the inflammatory macrophages in the process of liver fibrosis (44). It has been reported that the aberrant activated TGF-β/Smad signal pathway is the key link on the activation of HSCs and plays a crucial role in promoting myofibroblast differentiation, stimulates the production of ECM, and is involved in the development of hepatic fibrosis (37). Macrophages show the ability to regulate the secretion and activation of TGF-β, predominantly as TGF-β1, and produce excessive extracellular matrix. Meanwhile, once it binds to its receptors, TGFBR1 and TGFBR2, the proinflammatory macrophage is also activated by the upregulated expression of cell surface FcγRIII (45). TGF-β could also regulate the function of macrophages by limiting the production of iNOS and IFN-γ. Our data indicated that miR-130a-3p suppressed both the mRNA and protein expression of TGFBR1 and TGFBR2. By contrast, the expression of TGFBR1 and TGFBR2 could be restored by Antagomir-130a-3p. In addition, we also showed that miR-130a-3p enabled to bind to TGFBR1 and TGFBR2. These evidences suggested that miR-130a-3p could inhibit TGF-β/Smads signaling, at least in part, *via* TGFBR1 and TGFBR2 to affect the activation of HSCs and the phenotype of macrophages involving the development of liver fibrosis.

**Figure 8:**
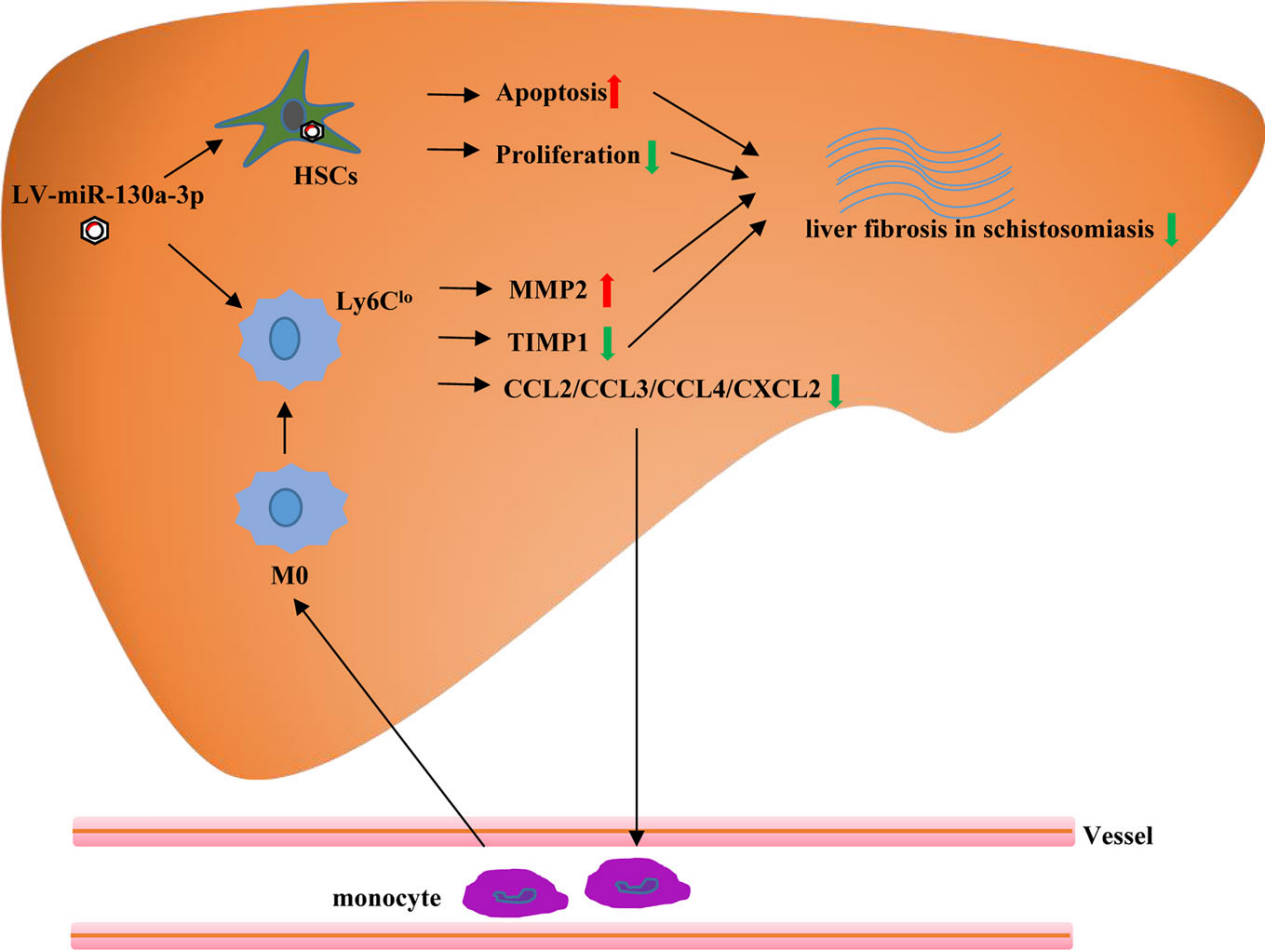
The graphical abstract of probable cellular and molecular mechanisms by which miR-130a-3p delivery mitigates liver fibrosis of schistosomiasis. Overexpression of miR-130a-3p increases HSC apoptosis and reduces proliferation and activation of HSCs. Moreover, miR-130a-3p could also upregulate the expression of Ly6C^lo^ macrophages, concomitant with decreased expression of TIMP1, and increased expression of MMP2, contributing to the dissolution of collagen.

In conclusions, our study demonstrated that the upregulated miR-130a-3p could ameliorate granulomatous inflammation in schistosomiasis liver fibrosis by inducing ;Ly6C^lo^-prone macrophages ;immune response, concomitant with the decreased expression of TIMP 1 and increased expression of MMP 2. Moreover, we found that LV-miR-130a-3p could enter into the HSCs and induce apoptosis and reduce proliferation and activation of HSCs (**Figure 8**). Hence, liver-targeting delivery of miR-130a-3p may be considered as a potential therapeutic option for the treatment of liver fibrosis in schistosomiasis.

## Data Availability Statement

The raw data supporting the conclusions of this article will be made available by the authors, without undue reservation.

## Ethics Statement

The Institutional Ethical Committee of Soochow University and Hefei Second Hospital approved the study, and all experiments were conducted in accordance with the principles of the Declaration of Helsinki including any relevant details. The patients/participants provided their written informed consent to participate in this study. The animal study was reviewed and approved by Institutional Animal Care and Use Committee (IACUC) of Soochow University for the use of laboratory animals (Permit Number: 201604A136).

## Author Contributions

LL, PW, and C-MX designed and conducted the experiments. LL, PW, and Y-SW contributed to development of methodology. LL, PW, Y-NZ, and CL contributed to analysis and interpretation of data. LL, PW, Z-YY, and Z-HL prepared figures and wrote the manuscript. T-ZZ, JX, and C-MX supervised the project and edited the manuscript. All authors contributed to the article and approved the submitted version.

## Funding

The study was supported by grants from the National Natural Science Foundation of China (Nos. 81772216, 81860358, and 81902083) and the Priority Academic Program Development of Jiangsu Higher Education Institutions (YX13400214).

## Conflict of Interest

The authors declare that the research was conducted in the absence of any commercial or financial relationships that could be construed as a potential conflict of interest.

## Publisher’s Note

All claims expressed in this article are solely those of the authors and do not necessarily represent those of their affiliated organizations, or those of the publisher, the editors and the reviewers. Any product that may be evaluated in this article, or claim that may be made by its manufacturer, is not guaranteed or endorsed by the publisher.
