# Supplementary material for: MiR-130a-3p Alleviates Liver Fibrosis by Suppressing HSCs Activation and Skewing Macrophage to Ly6Clo Phenotype
Source: Front Immunol. 2021 Aug 5;12:696069. doi: 10.3389/fimmu.2021.696069 (PMC8375151; doi:10.3389/fimmu.2021.696069)

**MiR-130a-3p alleviates liver fibrosis** **by suppressing HSCs activation and skewing macrophage to Ly6C^lo^ phenotype**

Lei Liu^1^^#^, Peng Wang^2#^, Yun-Sheng Wang^3^, Ya-Nan Zhang^1^, Chen Li^1^, Zi-Yin Yang^1^, Zi-Hao Liu^1^, Ting-Zheng Zhan^4^, Jing Xu^1^*, Chao-Ming Xia^1^*

**Supplementary Figure legends**Supplementary Fig. 1 The construction of liver fibrosis model of *S. japonicum* infection.

The infected mice were infected with 14 ± 1 cercariae of *S. japonicum* through abdominal skin. After 8 weeks, the liver tissue and serum between *S. japonicum*-infected group and non-infected group were collected. Liver tissue section was stained with H&E or Masson, respectively, and the original magnification of stained liver sections was 100×. The quantitative analysis was used for the evaluation of liver granulomas (A) or liver fibrosis (B). Sera HA was assayed by enzyme-linked immunosorbent assay (C). Liver HYP was measured by alkaline lysis method (D).

Supplementary Fig. 2 The identification of HSCs purity and the efficiency of LV-miR-130a-3p transfection.

We injected LV-miR-130a-3p or PBS into mice through the tail vein. After 72h, the HSCs were isolated by using density gradient centrifugation and cultured for 48h. Then the cells were identified the purity of HSCs by GFAP+ cell (A) and observed the efficiency of LV-miR-130a-3p entered HSCs under fluorescence microscope (B).

Supplementary Fig. 3 The transfection efficiency of JS1 cells.
JS1 cells were transfected with PBS, or Agomir-130a-3p/Agomir NC or Antagomir-130a-3p/Antagomir NC, respectively. Fluorescence microscope was conducted to observe whether cell transfection was effective.

Supplementary Fig. 4 The protein expression of α-SMA and Col I.

The protein expression of α-SMA and Col I were quantified and normalized by gray analysis (A, B).

Supplementary Fig. 5 The protein expression of ERK1/2, TGFBR1 and TGFBR2.

The protein expression of ERK1/2, TGFBR1 and TGFBR2 were quantified and normalized by gray analysis (A-C).

Supplementary Fig. 6 The mRNA expression of TGF-bR1, TGFbR2, and MAPK1 in the liver.

The liver tissues were collected from LV-miR-130a-3p, LV-NC or PBS group. The mRNA levels of target genes TGFBR1, TGFBR2 and MAPK1 were measured by qRT-PCR (A-C).

**Supplementary Fig. 1**


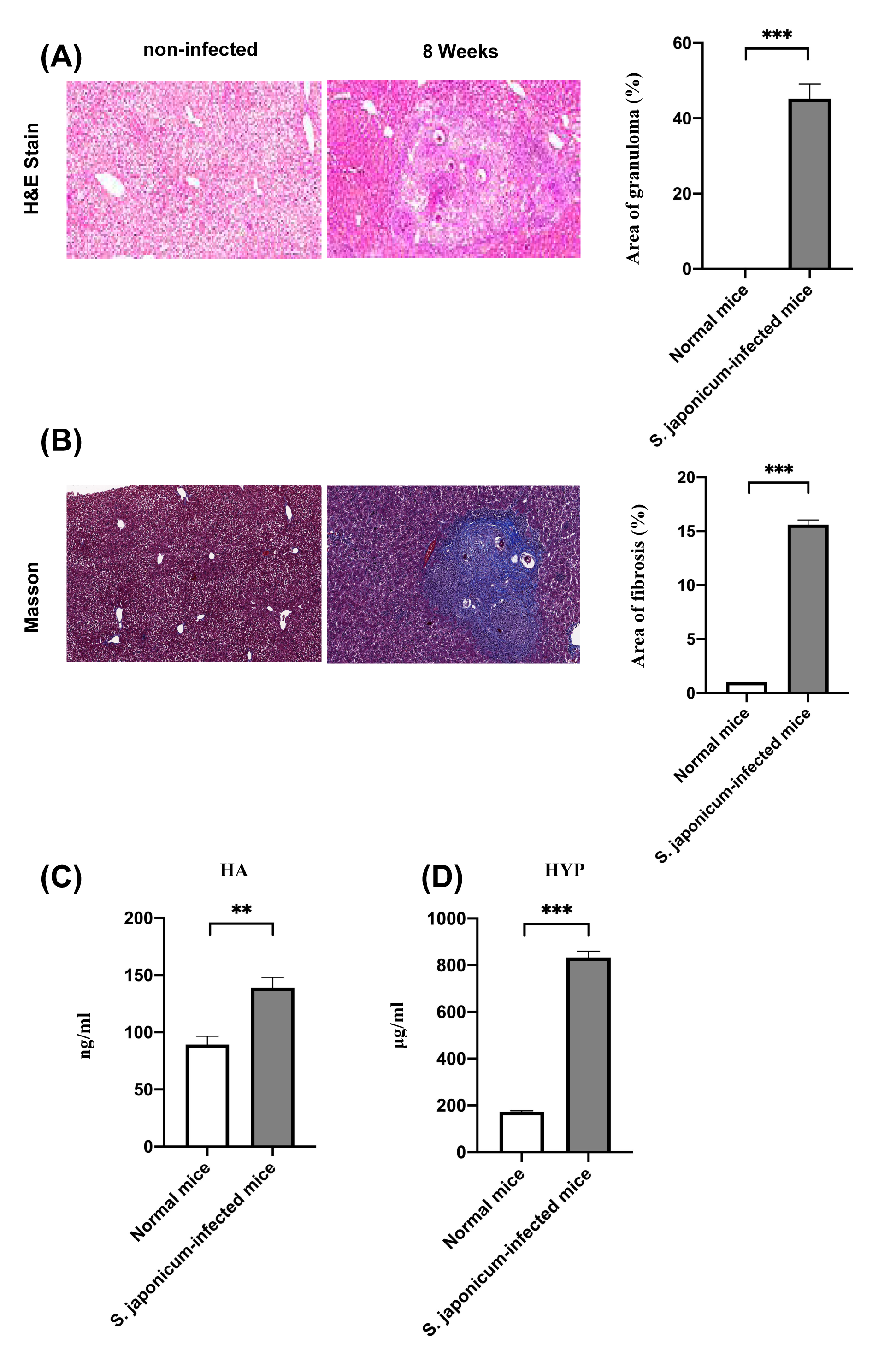


**Supplementary Fig. 2**


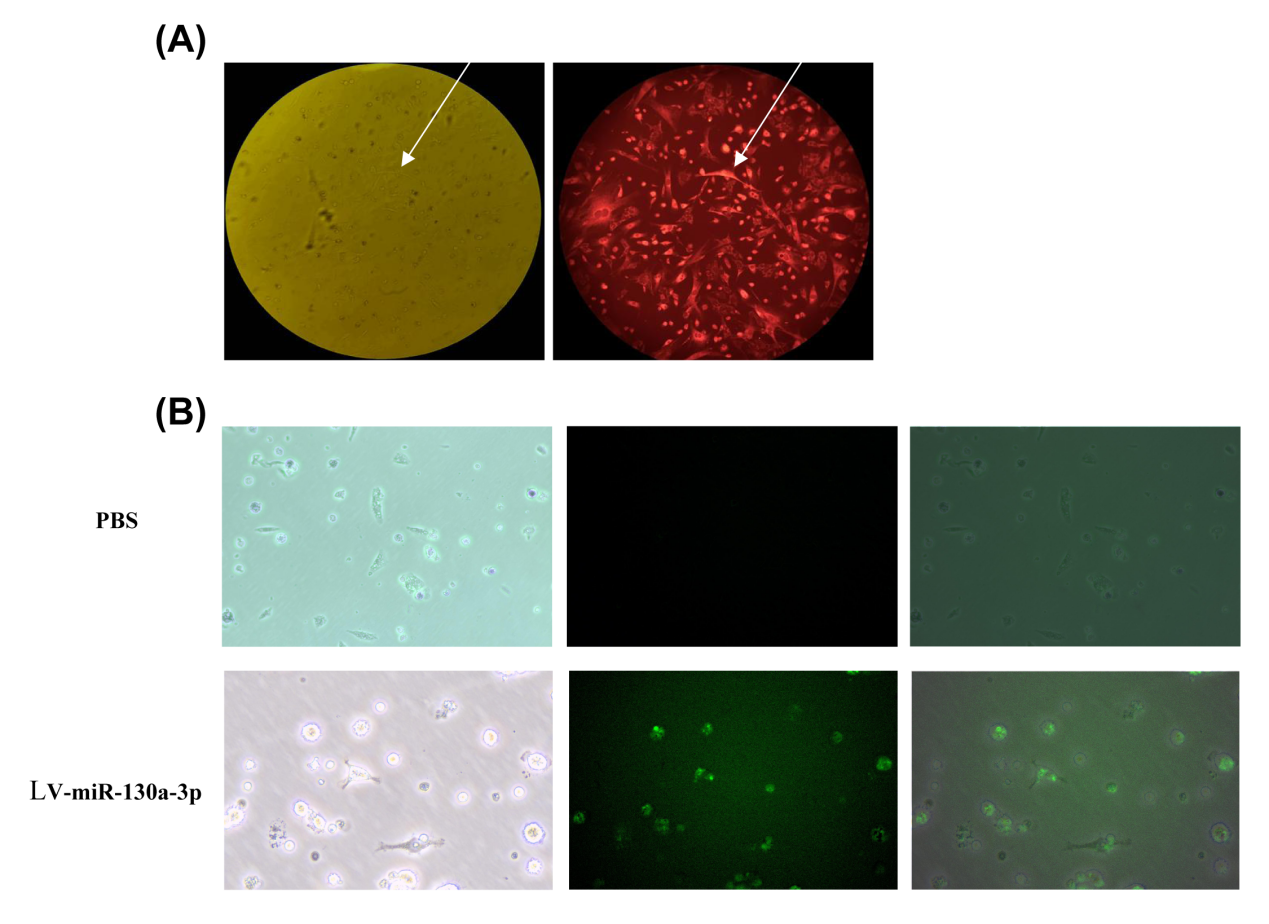


**Supplementary Fig. 3**


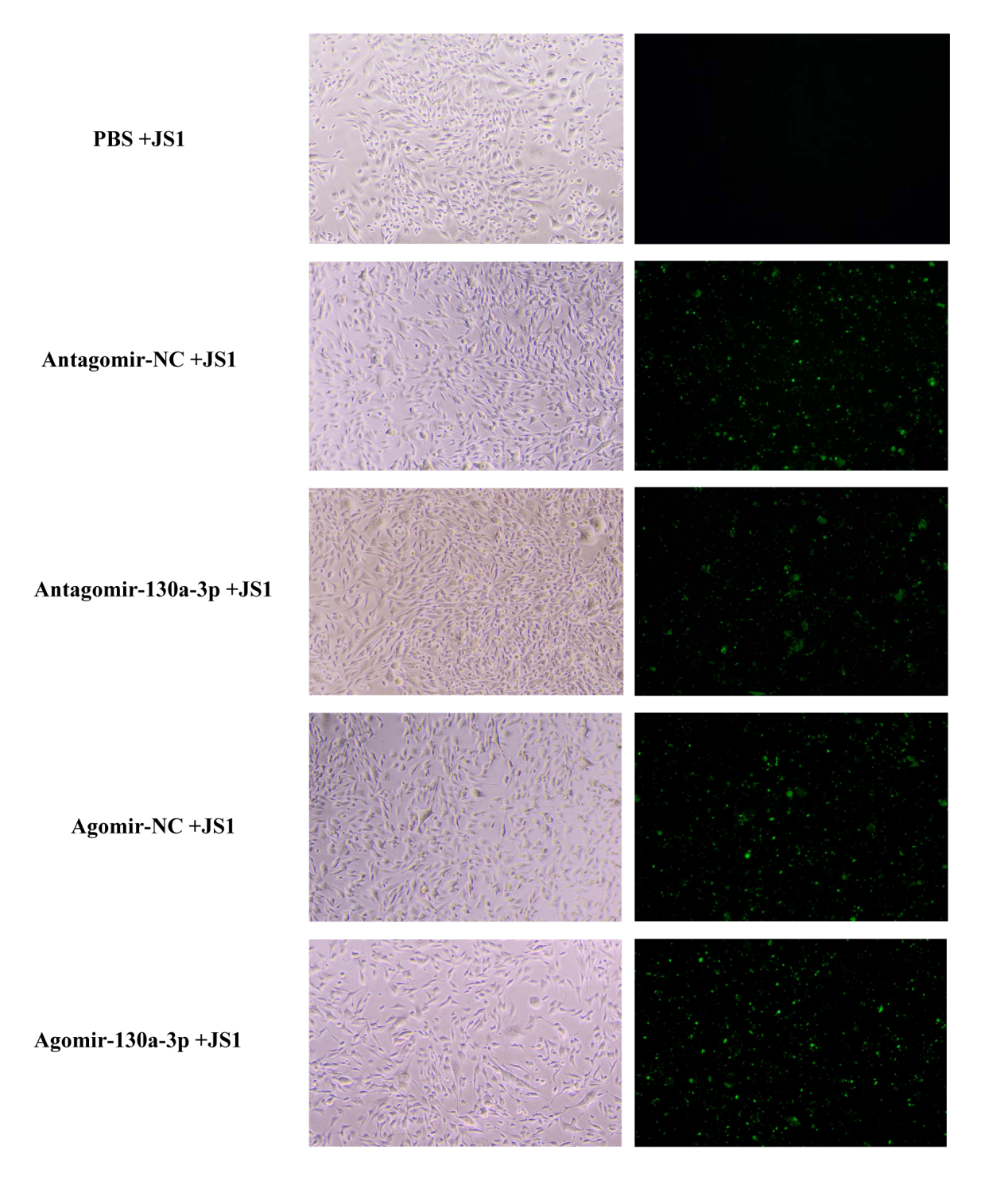


**Supplementary Fig. 4**


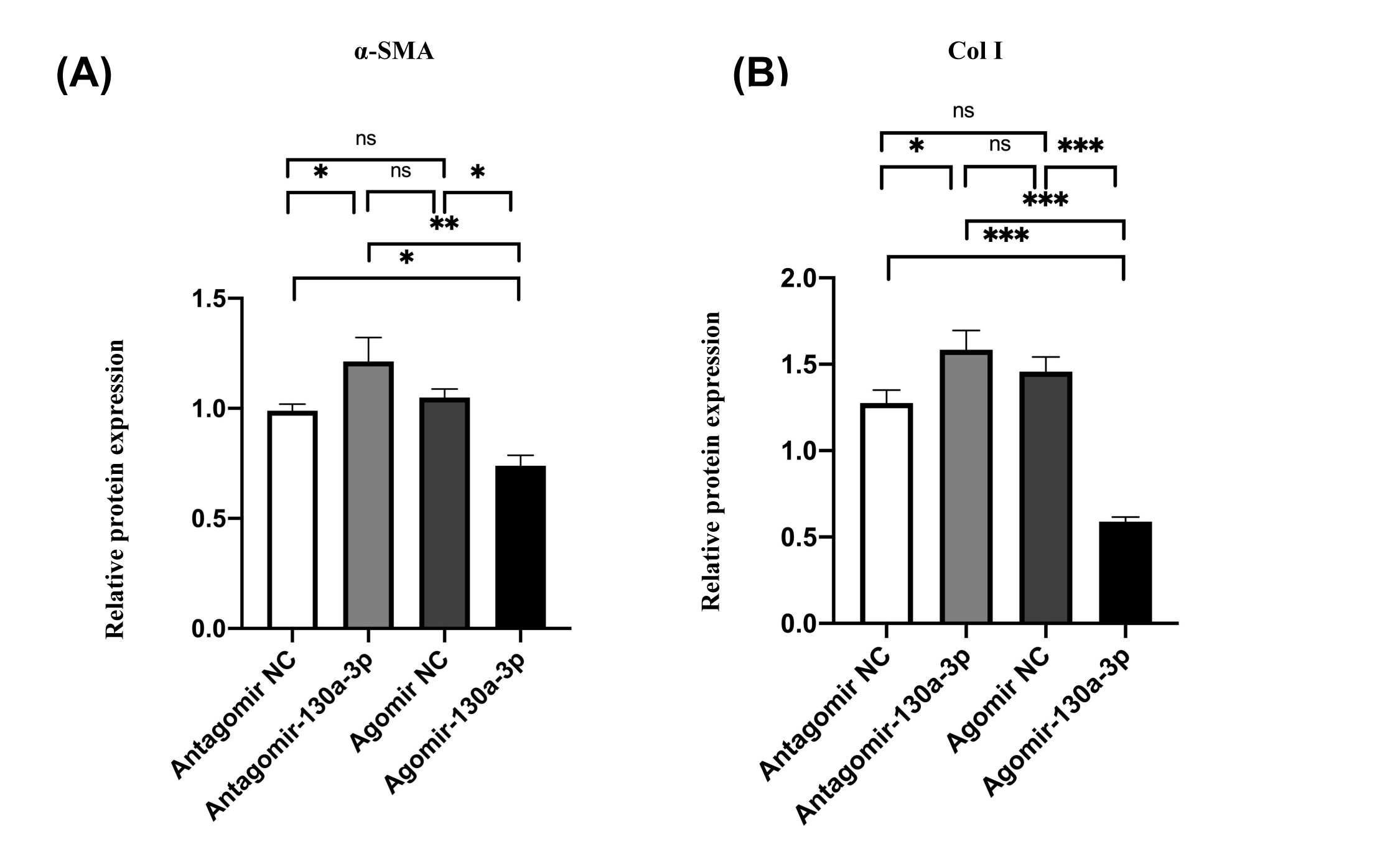


**Supplementary Fig. 5**


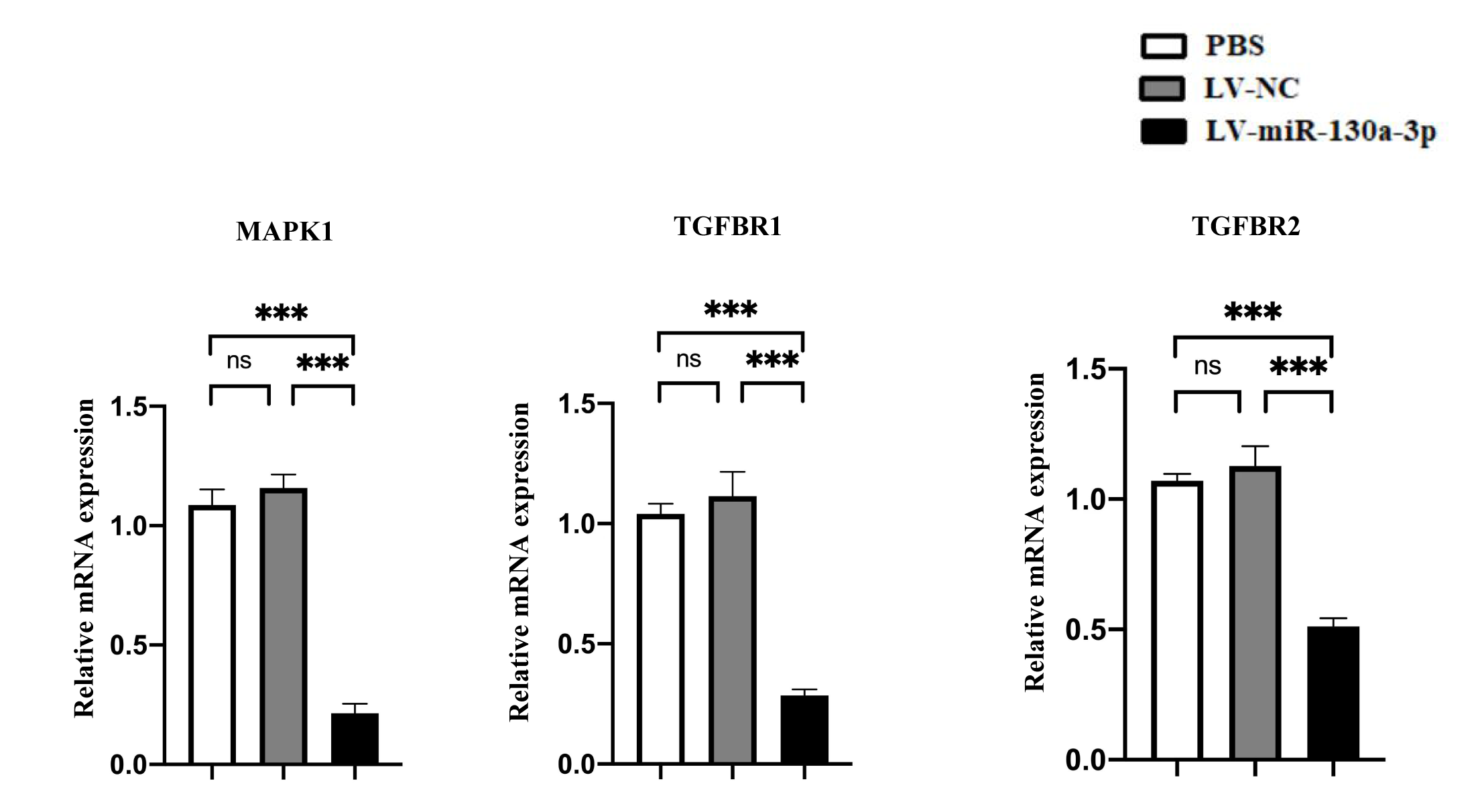


**Supplementary Fig. 6**


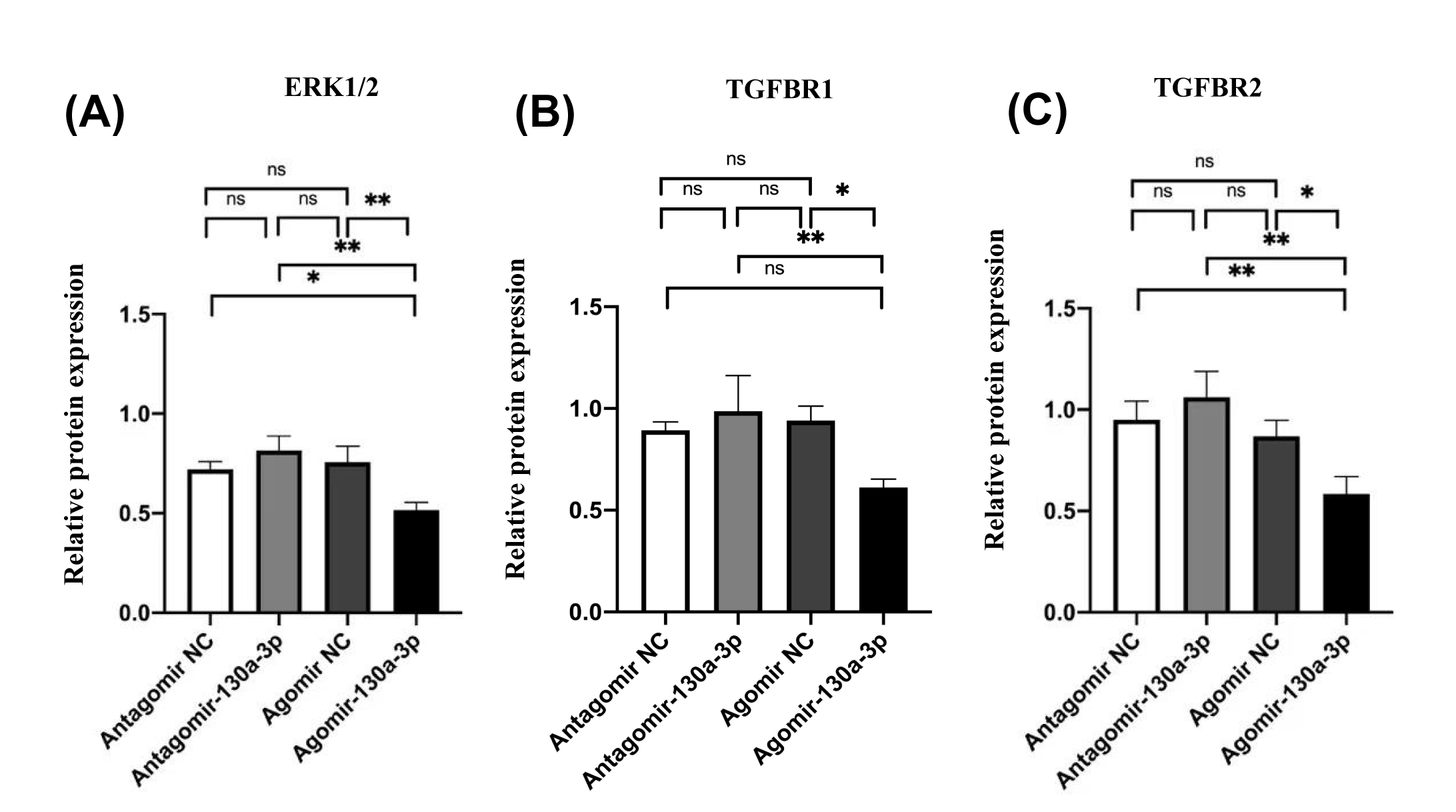

Supplement: Supplementary file 1 [file DataSheet_1.docx]
